# In pancreatic cancer patients, chemotherapy reshapes the gene expression profile and antigen receptor repertoire of T lymphocytes and enhances their effector response to tumor-associated antigens

**DOI:** 10.3389/fimmu.2024.1427424

**Published:** 2024-08-08

**Authors:** Silvia Brugiapaglia, Sara Bulfamante, Claudia Curcio, Maddalena Arigoni, Raffaele Calogero, Lisa Bonello, Elisa Genuardi, Rosella Spadi, Maria Antonietta Satolli, Donata Campra, Daniele Giordano, Paola Cappello, Francesca Cordero, Francesco Novelli

**Affiliations:** ^1^ Department of Molecular Biotechnology and Health Sciences, University of Turin, Turin, Italy; ^2^ Molecular Biotechnology Center “Guido Tarone”, University of Turin, Turin, Italy; ^3^ Centro Oncologico Ematologico Subalpino, Azienda Ospedaliera Universitaria (A.O.U.) Città della Salute e della Scienza di Torino, Turin, Italy; ^4^ Struttura Complessa (SC) Chirurgia generale d’urgenza e pronto soccorso, Azienda Ospedaliera Universitaria (A.O.U.) Città della Salute e della Scienza di Torino, Turin, Italy; ^5^ Computer Science Department, University of Turin, Turin, Italy

**Keywords:** pancreatic cancer, chemotherapy, tumor-associated antigens, T lymphocyte response, anti-tumor responses

## Abstract

**Introduction:**

Pancreatic Ductal Adenocarcinoma (PDA) is one of the most aggressive malignancies with a 5-year survival rate of 13%. Less than 20% of patients have a resectable tumor at diagnosis due to the lack of distinctive symptoms and reliable biomarkers. PDA is resistant to chemotherapy (CT) and understanding how to gain an anti-tumor effector response following stimulation is, therefore, critical for setting up an effective immunotherapy.

**Methods:**

Proliferation, and cytokine release and TCRB repertoire of from PDA patient peripheral T lymphocytes, before and after CT, were analyzed in vitro in response to four tumor-associated antigens (TAA), namely ENO1, FUBP1, GAPDH and K2C8. Transcriptional state of PDA patient PBMC was investigated using RNA-Seq before and after CT.

**Results:**

CT increased the number of TAA recognized by T lymphocytes, which positively correlated with patient survival, and high IFN-γ production TAA-induced responses were significantly increased after CT. We found that some ENO1-stimulated T cell clonotypes from CT-treated patients were expanded or de-novo induced, and that some clonotypes were reduced or even disappeared after CT. Patients that showed a higher number of effector responses to TAA (high IFN-γ/IL-10 ratio) after CT expressed increased fatty acid-related transcriptional signature. Conversely, patients that showed a higher number of regulatory responses to TAA (low IFN-γ/IL-10 ratio) after CT significantly expressed an increased IRAK1/IL1R axis-related transcriptional signature.

**Conclusion:**

These data suggest that the expression of fatty acid or IRAK1/IL1Rrelated genes predicts T lymphocyte effector or regulatory responses to TAA in patients that undergo CT. These findings are a springboard to set up precision immunotherapies in PDA based on the TAA vaccination in combination with CT.

## 1 Introduction

Pancreatic Ductal Adenocarcinoma (PDA) is the most frequent neoplasia that affects the pancreas, and its incidence is increasing, making it the third cause of cancer death (1). PDA remains an almost incurable tumor with a 5-year overall survival rate of just 13% (1). This poor prognosis is due to the lack of early symptoms, which restricts the percentage of patients that can undergo surgery to 20% (2). Surgical resection is the main treatment to achieve long-term survival since PDA is mostly resistant to chemotherapy (CT). In the last decade, however, other chemotherapeutic agents, such as FOLFIRINOX and nab-paclitaxel, have been administered instead of, or in combination with, the standard drug gemcitabine (GEM), leading to a slight increase in survival of PDA patients (3–5).

The main reason for chemoresistance is the composition of the tumor microenvironment (TME) rich in fibroblasts and pancreatic stellate cells, which produce the extracellular matrix elements (6, 7). Moreover, the PDA TME is poorly populated by leukocyte cells and in advanced cancer most of them are represented by immunosuppressive cells, such as tumor-associated macrophages (TAM), myeloid-derived suppressor cells (MDSC), and regulatory T cells (Treg) (8). Only a few effector T lymphocytes infiltrate the tumor with no evidence of activation (8).

PDA is considered a “cold” tumor due to its immunosuppressive state and the low rate of DNA mutations, which does not generate enough neoantigens that can be recognized by the immune system (9); research is thus increasingly directed toward strategies that can trigger and enhance the immune response to cancer cells. One of the most studied immunotherapeutic approaches is tumor vaccination; several clinical trials have exploited vaccination to elicit the immune response, using a peptide of an overexpressed or mutated protein, such as Mucin 1 (10), telomerase (11), survivin (12), or KRAS (13); or with whole tumor cells, such as GVAX, which is composed of granulocyte-macrophage colony-stimulating factor (GM-CSF)–secreting tumor cells (14, 15). Moreover, DNA vaccination targeting the overexpressed glycolytic enzyme alpha-enolase (ENO1), alone and much more in combination with CT or with MDSC inhibitor, efficiently delays tumor growth in vaccinated mice that spontaneously develop PDA (16–18).

In a first cohort of PDA patients, we previously showed that CT enhanced the antibody response of PDA patients against some TAA, including ENO1, far upstream binding protein 1 (FUBP1), keratin type II cytoskeletal 8 (K2C8) and glyceraldehyde-3-phosphate dehydrogenase (GAPDH) (17, 19). In this study we investigate the immunological tone, TCRB clonotype repertoire and transcriptional profile in a second cohort of PDA patients. We found that CT profoundly reshaped TAA-stimulated T lymphocyte responses toward an effector phenotype, which is associated with spreading of TCR clonotypes and specific gene expression profiles.

## 2 Materials and methods

### 2.1 Patients

From 2005 to 2011, 23 patients with PDA (**Tables 1**, **2**) were enrolled in this study and provided sera and peripheral blood mononuclear cells (PBMC). The protocol was approved by the local research ethical committee (Azienda Ospedaliero-Universitaria Città della Salute e della Scienza di Torino, IT) and investigations were performed according to the Helsinki Declaration principles. All participants in the study signed a declaration of informed consent. PBMC samples were collected from venous blood before and after one round of CT, consisting of three or six cycles of drug infusion. PBMC were separated by Ficoll-Hipaque (Sigma-Merck, Darmstadt DE), frozen and stored in liquid nitrogen until required for use.

**Table 1:** Clinical data of PDA patients.

Patient	Gender	Age	Surgery	Stage	M	CT	Response	OS
**6**	F	69	Yes	III	No	GEM	PD	5.2
**7**	F	69	No	IIB	No	GEM	PD	3.9
**11**	M	54	No	IV	Yes	GEM + 5FU	PD	9.5
**16**	F	51	No	IV	Yes	GEM	PD	2.2
**20**	M	59	No	IV	Yes	GEMOX	SD/PR	11.8
**24**	F	74	No	IV	Yes	GEM	PD	9.8
**29**	M	59	No	III	No	GEM	PD	13.4
**31**	F	70	No	IV	Yes	GEM	PD	3.4
**32**	F	73	Yes	IIB	No	GEM	SD/PR	11.9
**35**	M	62	No	IV	Yes	GEM	SD/PR	8.2
**37**	F	67	Yes	IV	Yes	GEM	PD	11.8
**50**	M	46	Yes	IIB	No	GEM	SD/PR	20.3
**51**	F	49	No	IV	Yes	GEM	PD	3.1
**83**	M	55	Yes	IIB	No	GEM	SD/PR	51.7
**85**	F	64	No	III	No	BEV+RT+CAP	SD/PR	35.7
**143**	F	79	No	III	No	GEM	SD/PR	15.0
**158**	M	55	No	IIB	No	GEM	PD	10.3
**184**	F	73	Yes	IV	Yes	GEM	PD	9.7

M, Metastasis; GEM, Gemcitabine; 5FU, 5-fluorouracil; GEMOX, GEM + Oxaliplatin; BEV, Bevacizumab; RT, radiotherapy; CAP, capecitabine; PD, progression disease; SD/PR, stable disease/partial response; OS, overall survival (months).

Patient ID is indicated in bold.

**Table 2:** Clinical data of PDA patients.

Patient	Gender	Age	Surgery	Stage	M	CT	Response	OS
**32**	F	73	Yes	IIB	No	GEM	SD/PR	11.9
**163**	M	63	Yes	IIB	No	GEMOX	PD	12.4
**186**	F	73	Yes	IV	Yes	GEM	PD	34.3
**10**	F	74	No	IV	Yes	GEMOX	PD	16.4
**99**	M	69	No	IV	Yes	Folfirinox	PD	9.1
**117**	F	74	No	III	No	GEM+other	PD	15.2

M, Metastasis; GEM, Gemcitabine; GEMOX, GEM + Oxaliplatin; PD, progression disease; SD/PR, stable disease/partial response.

Patient ID is indicated in bold.

### 2.2 PBMC culture

PBMC were thawed and cultured at 5x10^5^ cells/ml in 96-well round-bottomed plates with RPMI (Gibco-ThermoFischer, Waltham MA) plus 5% certified FBS (Gibco-ThermoFischer, Waltham MA) in the presence or absence of 5 µg/ml for each recombinant protein, namely ENO1 (Sigma-Merck, Darmstadt DE), FUBP1, K2C8, and GAPDH (Origene, Rockville, MD).

### 2.3 Proliferation

At 4 days of culture, BrdU was added at a final concentration of 10 μM. After 18 h, the specific proliferation of TAA-stimulated PBMC was evaluated by Cell Proliferation ELISA BrdU incorporation, following the manufacturer’s instructions (Roche-Merck Darmstadt DE). Optical Density (O.D.) at 450 nm (reference wavelength: 690 nm) was detected with VICTOR^®^ Nivo™ Multimode Microplate Reader (PerkinElmer, Waltham, MA). Stimulation Index (S.I.) is the O.D. ratio between the BrdU incorporation in the presence of TAA and BrdU incorporation in control medium condition. When the S.I. was ≥ 2 the response to TAA was considered as a “proliferative response”.

### 2.4 Cytokine evaluation

At 3 days of culture, supernatants from TAA-stimulated PBMC were collected and stored at -20°C until required for use. Supernatants were diluted at 1:4 in assay diluent and tested in ELISA for the presence of IFN-γ, IL-10, IL-12(p70) and IL-17A following the manufacturer’s instructions (Biolegend, San Diego, CA). O.D. at 450 nm (reference wavelength: 570 nm) was detected with VICTOR^®^ Nivo™ Multimode Microplate Reader (PerkinElmer, Waltham, MA). The IFN-γ production by TAA-stimulated PBMC was arbitrarily categorized as null (no detection), low (< 100 pg/ml), intermediate (≥ 100 < 1000 pg/ml) and high (≥ 1000 pg/ml).

### 2.5 TCRB sequencing

PBMC were thawed and cultured in the presence of ENO1, as previously described. At 5 days, PBMC were harvested, pelleted, and processed for mRNA extraction following the manufacturer’s instructions with Maxwell^®^ RSC simplyRNA Cells Kit compatible with Maxwell^®^ RSC Instrument (Promega, Madison, WI). The concentration of mRNA was evaluated with QuantiFluor^®^ RNA System (Promega, Madison, WI). RT-PCR was performed with SuperScript™ III Reverse Transcriptase (Invitrogen-ThermoFischer, Waltham MA) to obtain cDNA; cDNA concentration was assumed to be the same as the mRNA used. Manufacturer’s instructions of LymphoTrack^®^ TRB Assay – MiSeq^®^ (Invivoscribe, San Diego, CA) were followed to generate the library pool, which was sequenced with MiSeq™ System (Illumina, San Diego, CA) using the MiSeq Reagent Nano Kit v2 (500-cycles) (Illumina, San Diego, CA).

### 2.6 RNA-Seq

PBMC were thawed and processed for RNA extraction following the manufacturer’s instructions with Maxwell^®^ RSC simplyRNA Cells Kit compatible with Maxwell^®^ RSC Instrument (Promega, Madison, WI). RNA concentration and quality were estimated with NanoVuePlus Spectrophotometer (GE Healthcare) and Agilent 2100 Bioanalyzer (Agilent Technologies), respectively. Libraries for RNA sequencing were generated using TruSeq RNA Access sample preparation kit v2 (Illumina Inc., San Diego, CA, USA) following the manufacturer’s instructions, using 150 ng of total RNA as input material. Libraries were pooled at equi-molar concentrations, quantified with Qubit DNA HS kit (ThermoFisher) and run at the concentration of 1.7 pM on the NextSeq500 sequencer (Illumina Inc.) in 75 nts paired end sequencing mode following the manufacturer’s instructions.

### 2.7 Immunological tone

For hierarchical clustering analysis of the IFN-γ/IL10 ratio, data were log2 transformed, normalized and clustered using the Euclidean distance as the similarity metric.

### 2.8 Bioinformatic analysis of TCRB repertoire

Analysis of TCRB sequencing was performed with the online tool IMGT^®^ System starting from the fastq files and a list of unique clonotypes was generated. Each clone, characteristically different, is indicated as a “clonotype” and corresponds to a specific V(D)J rearrangement; of note, more than one clonotype can correspond to the same V(D)J rearrangement. The frequency of each clonotype present with ENO1 stimulation (ENO1) was compared to the frequency measured in the basal condition (medium) by subtracting the clonotype proportion. Only the clonotypes with a significant variation were considered. Only clonotypes with >2 counts were considered in the subsequent analysis. Normalized frequency = no. of clonotype counts/no. of total counts x 10000. The difference in frequencies between ENO1 and medium was statistically analyzed with the Chi-squared test.

### 2.9 Differential expression analysis of RNA expression profiles

The analysis of fastq files utilized the docker4seq package (20). Assessment of fastq file quality was conducted using fastqc. Skewer was employed for trimming fastq files, and STAR 2.5 was used for mapping against the human genome version hg38. Subsequently, differential expression analysis was carried out through DEseq2 (21). The identified differentially expressed genes (DEGs) underwent filtration based on specified thresholds for recorded fold changes (|log2FC| ≥ 1) and statistical significance (p < 0.05).

### 2.10 Immune cell composition and gene correlation analysis

The estimation of immune cell types was conducted using CIBERSORTx, an analytical tool employed to estimate the abundance of cell types by analyzing gene expression data (22). In this study, CIBERSORTx was applied to the RNAseq data to determine the relative proportions of immune cell types. Subsequently, Spearman correlation analysis was performed between the proportions of immune cell types and gene expression levels.

All figures and statistical analyses were performed using R 4.3.2 version. All the sequencing data has been deposited here: 10.6084/m9.figshare.25975231 and BioProject ID PRJNA1144662.

## 3 Results

### 3.1 Effect of CT on PDA patient T lymphocyte responses to TAA

The proliferation and IFN-γ production in response to *in vitro* stimulation with four TAA (ENO1, FUBP1, K2C8 and GAPDH) were analyzed in peripheral T lymphocytes obtained from PDA patients before and after CT. PDA patients were enrolled at the time they were starting Gemcitabine (GEM)–based CT; clinical data of patients are reported in **Table 1**, patients were analyzed after only one round of CT, as most patients discontinued CT treatment because of their poor performance status or due to their demise.

**Figure 1:**
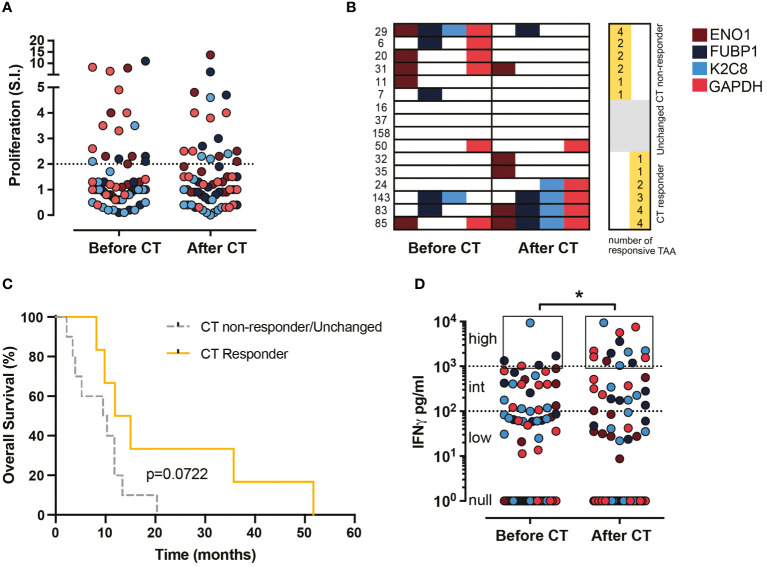
Effect of CT on proliferation and IFN-γ production of TAA-stimulated PBMC from PDA patients before and after CT. **(A)** Proliferative response (S.I. ≥ 2) to TAA of PBMC from 16 patients before and after CT (64 responses were described). **(B)** Representation of the proliferative response to the four TAA (ENO1, FUBP1, K2C8 and G3P) by each patient before and after CT. Patients were categorized into two groups: CT responder and CT non-responder/Unchanged. **(C)** Survival curves of CT responder patients (yellow) and CT non-responder/Unchanged (gray) groups. **(D)** IFN-γ levels (pg/ml) in supernatants of TAA-activated PBMC from 18 patients before and after CT (72 responses were described). Null responses: no detection, low responses: < 100 pg/ml, intermediate responses: ≥ 100 < 1000 pg/ml, high responses: ≥ 1000 pg/ml. Brown dots refer to ENO1, blue dots to FUBP1, light blue dots to K2C8 and red dots to GAPDH. Statistics were calculated by the Chi-square test on the percentage of high responses before and after CT. * = P<0.05.

The *in vitro* proliferative response (S.I. >2) of ENO1, FUBP1, K2C8 or GAPDH-stimulated T lymphocytes from 16 PDA patients before and after CT was evaluated. Out of the 64 expected responses (100%), the percentage of proliferative responses was similarly observed before and after CT (28%) (**Figure 1A**). Considering the number of proliferative responsive TAA in each PDA patient, three categories were identified: those in whom i) the number decreased after CT (CT non-responder), ii) the number of recognized TAA was not changed before or after CT (unchanged); iii) the number increased after CT (CT responder) (**Figure 1B**). Out of 16 patients, 25% (4/16) showed null or the same number of recognized TAA before and after CT, 37% (6/16) showed an increased number of recognized TAA after CT, and the other 37% (6/16) showed a decreased number of recognized TAA after CT (**Figure 1B**). The overall survival of “CT responders” was higher than that of “CT non-responders/unchanged”, even if it was not statistically significant (**Figure 1C**).

To assess the impact of CT on the anti-tumor effector response of patient T lymphocytes following stimulation with ENO1, FUBP1, K2C8 or GAPDH, the secretion of IFN-γ was evaluated. The percentage of high IFN-γ production (> 1000 pg/ml) increased significantly (from 6.9 to 18.1%) after CT compared to before CT, while no differences were found in the percentage of intermediate (>100<1000 pg/ml) and low (< 100 pg/ml) IFN-γ responses (**Figure 1D**). These data indicated that CT induces a high production of IFN-γ rather than proliferation in response to TAA stimulation in T lymphocytes from PDA patients.

### 3.2 Effect of CT on the immunological tone of T lymphocytes in response to TAA in PDA patients

**Figure 2:**
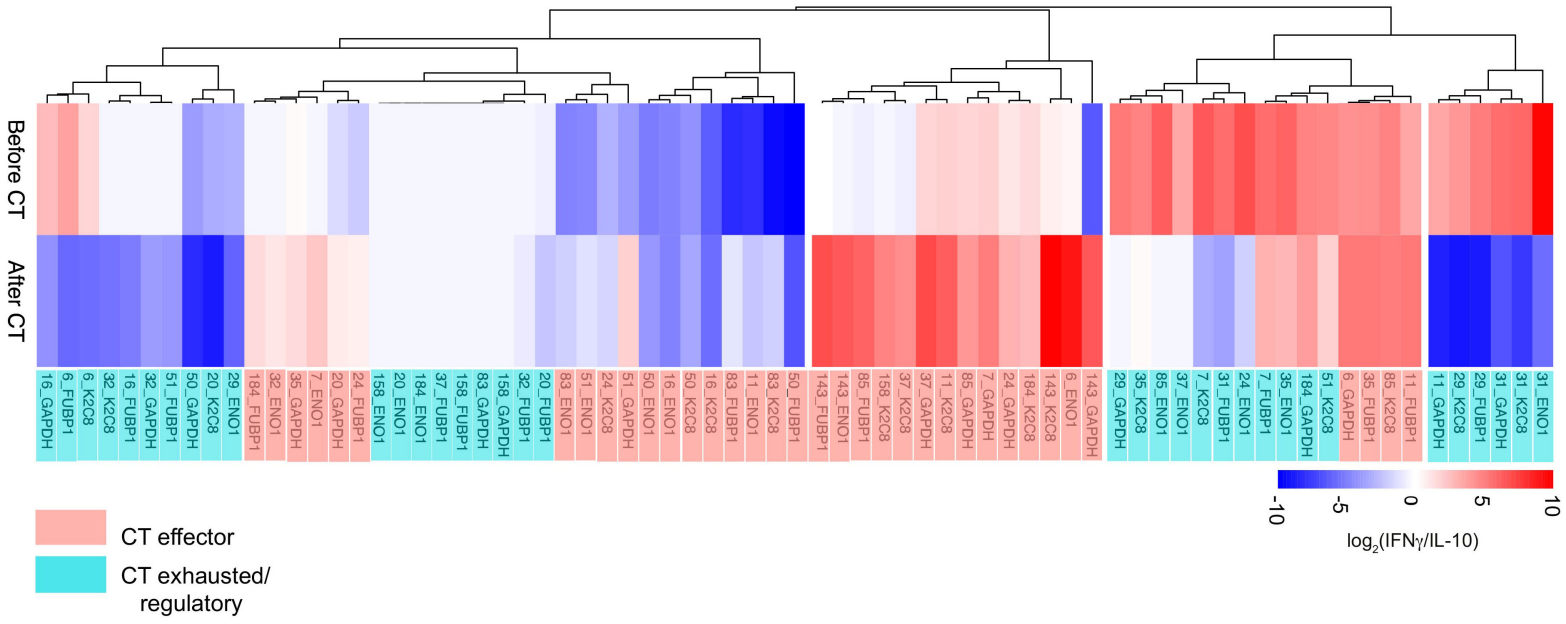
Hierarchical clustering analysis of IFN-γ/IL10 ratio of TAA-stimulated PBMC from 18 PDA patients before and after CT. The effector-like response is indicated in red with the IFN-γ/IL10 ratio > 1, while the regulatory-like response is indicated in blue and characterized by the IFN-γ/IL10 ratio < 1. CT effector responses refer to the CT-dependent shift of the ratio toward a higher IFN-γ production or a lower IL-10 (light red). CT exhausted/regulatory responses refer to the CT-dependent shift toward a lower IFN-γ production, a higher IL-10 production or unchanged response (light blue).

To evaluate the immunological tone, IFN-γ and IL-10 production was measured from the four TAA-stimulated T lymphocytes from 18 PDA patients, obtaining 72 total TAA-specific responses. The ratio between IFN-γ and IL-10 levels was represented as a heatmap according to the clustering analysis (**Figure 2**). When IFN-γ production exceeds IL-10 production, the tone is considered as effector-like, whereas the regulatory-like tone refers to higher IL-10 production. Among the analyzed patients, 88% (16/18) showed at least one TAA-dependent effector response before or after CT.

Clustering analysis of the immunological tone identified two groups of T lymphocyte responses: “CT Effector”, which displayed an increased functional or a lower regulatory tone after CT (36/72; 50%) and “CT Exhausted/Regulatory”, which shifted their functional tone toward a regulatory one after CT or which displayed null cytokine production both before and after CT (36/72; 50%) (**Figure 2**). These results indicated that CT is able to shift 50% of the T lymphocyte responses toward an effector immunological tone.

In the same cohort of PDA patients, the production of additional cytokines relevant for the PDA immunological tone such as IL-12 and IL-17 was performed (**Supplementary Table 1**). The analysis of IL-12 and IL-17 secretion after CT revealed an increase in 13% and 21% of TAA-induced responses respectively, whereas only 7% and 9% of responses were decreased. These data indicate that about 17% of TAA-stimulated PBMC produced increased level of IL-12 and/or IL-17 after CT, endorsing the hypothesis that CT modulates the cytokine release toward Th1 and Th17-like response in a subset of patients.

### 3.3 Analysis of ENO1-specific TCRB clonotypes in PDA patients before and after CT

The TAA-induced clonotypic profile of peripheral T lymphocytes was investigated through the mRNA sequencing of T Cell Receptor Beta (TCRB) repertoire. The T lymphocytes, obtained from an additional cohort of PDA patients before and after CT (**Table 2**), were stimulated *in vitro* with ENO1.

**Figure 3:**
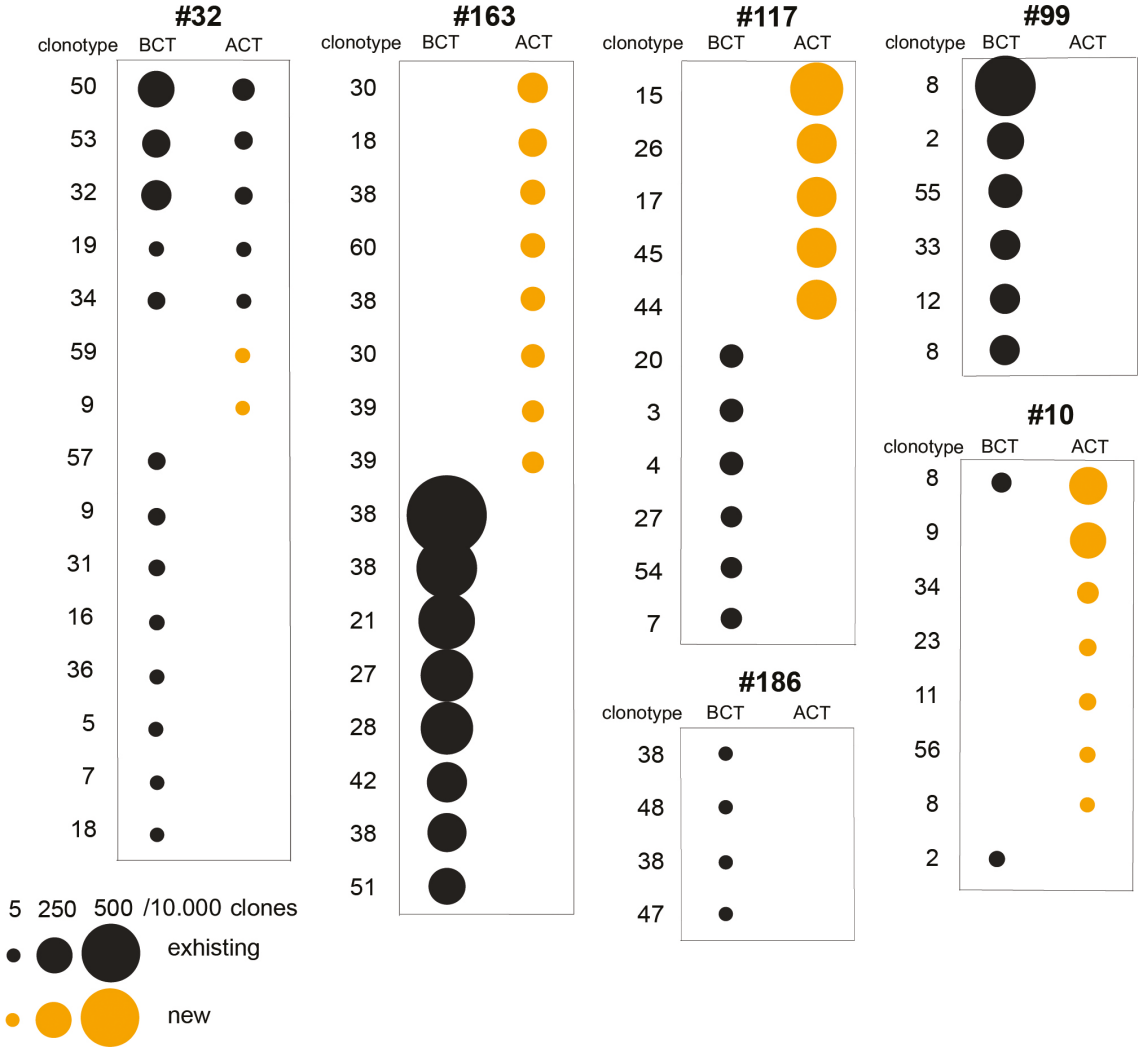
Analysis of TCRB repertoire and immunological tone of TAA-activated T cells from PDA patents before and after CT. T cell clonotypes significantly expanded following ENO1 *in vitro* stimulation before and after CT in six PDA patients. Clonotype numbers indicate the V(D)J rearrangement and each unique clonotype is defined by the CDR3 region, both listed in **Supplementary Table 2**. Clonotypes are represented by a circle whose size indicates the increase of ENO1-expanded clone per 10,000 clones. Black circles are existing clonotypes before CT and yellow circles are new ones after CT.

Before CT, all six PDA patients showed a variable number, mostly private, of ENO1-specific clonotypes (**Figure 3**). After CT, in all patients the majority of ENO1-specific clonotypes detected before CT disappeared, with a few that decreased or remained unchanged. Notably, in four out of the six patients (#32, #163, #117, #10) CT induced from two to eight new clonotypes (**Figure 3** and **Supplementary Table 2**).

### 3.4 Gene expression profile of PBMC in PDA patients before and after CT

To evaluate a possible link between the gene expression profile of PBMC and the immunological tone of TAA-induced T lymphocyte responses, the transcriptional state of PBMC collected before and after CT from ten PDA patients of the same cohort (**Table 1**) was investigated.

Analysis of the cellular composition, based on data from RNA samples analyzed through transcriptome deconvolution, revealed that the percentage of immune cell subsets of each PDA patient underwent modifications after CT (**Supplementary Figure 1**). However, CT did not reshape the immune components in a statistically significant manner when all patients were analyzed together (**Supplementary Figure 1**).

**Figure 4:**
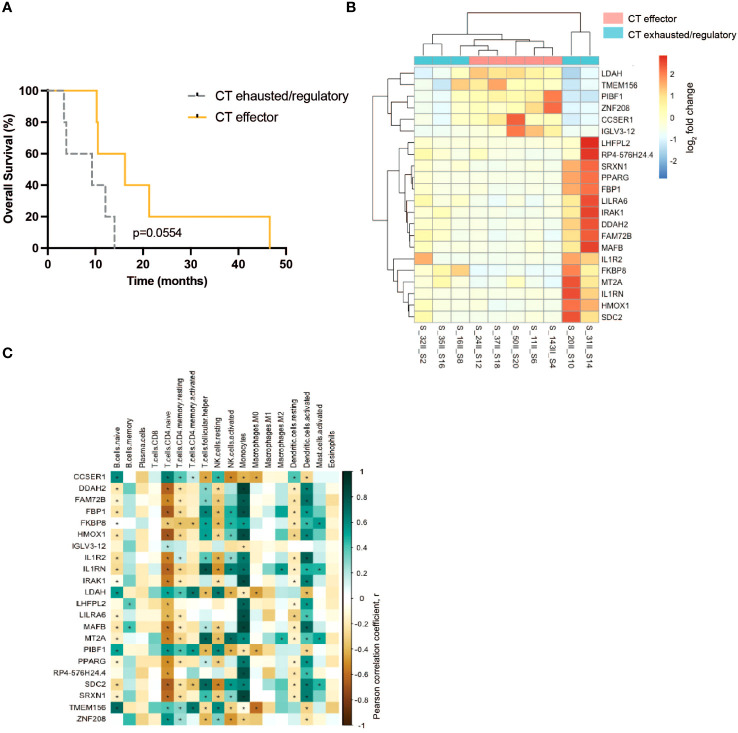
Analysis of the immuno-components and the transcriptional profile of PBMC from PDA patients before and after CT. **(A)** Survival curves of CT effector patients (yellow) and CT exhausted/regulatory (gray) groups. **(B)** Differential expressed genes between CT exhausted/regulatory (light blue) versus CT Responder (light red) patients after CT. **(C)** Correlation between the cell fraction of immune populations and the differential expressed gene levels. Positive correlation (green); negative correlation (brown). Statistical analysis was performed by Pearson correlation, p= 0.05. * = P<0.05.

To investigate whether gene expression predicts the effector/regulatory response to TAA of T lymphocytes of PDA patients before or after CT, differential gene expression (DGE) analysis was employed. DGE analysis did not show any significant differentially expressed genes between before and after CT. Nevertheless, this DGE analysis showed a number of differentially expressed genes when the PDA patient cohort was subdivided in two groups based on the number of T lymphocyte responses to TAA stimulation that shifted from regulatory to effector tone after CT. PDA patients that displayed a higher number of effector-like-shifted responses were grouped as “CT Effector”, whereas PDA patients that displayed a higher number of regulatory-like-shifted responses were grouped as “CT Exhausted/Regulatory” (**Supplementary Table 3**). Two patients (#16, #35) who displayed an equal number of effector and regulatory-like-shifted responses were included in CT Exhausted/Regulatory group because of the absence of predominant effector-like immunological tone (**Supplementary Table 3**). Notably, the “CT Effector” group showed a trend of increased survival compared to the “CT Exhausted/Regulatory” group (**Figure 4A**).

DGE analysis conducted on patient PBMC before CT did not identify differentially expressed genes. However, on patient PBMC after CT, DGE analysis revealed alterations in the expression of 22 genes (**Figure 4B**). The expression of six genes (LDAH, TMEM156, PIBF1, ZNF208, CCSER1, IGLV3–12) was higher in the “CT Effector” group, and the expression of the other 16 genes (LHFPL2, SIRPB1-SIRPD, SRXN1, PPARG, FBP1, LILRA6, IRAK1, DDAH2, FAM72B, MAFB, IL1R2, FKBP8, MT2A, IL1RN, HMOX1, SDC2) was higher in the “CT Exhausted/Regulatory” group (**Figure 4B**).

The gene expression level of the 22 differentially expressed genes was correlated to the abundance of specific immune cell subsets that express these genes, as identified by DGE analysis. Data showed a positive correlation (in green) between the expression of three genes (LDAH, PIBF1 and TMEM156) and the abundance of naive CD4 T cells, resting memory CD4 T cells, activated memory CD4 T cells and resting NK cells (**Figure 4C**). Conversely, a negative correlation (in brown) between the same three genes and the abundance of activated NK cells and dendritic cells was found (**Figure 4C**). Notably, the expression of IL1R pathway genes (IRAK1, IL1R2 and IL1RN) was positively correlated with the presence of activated NK cells, monocytes and dendritic cells (**Figure 4C**).

## 4 Discussion

We have previously demonstrated that, in PDA patients, CT induces an antitumoral immune response, both in terms of antibody production and effector T lymphocyte response against four overexpressed PDA-associated TAA, namely ENO1, FUBP1, K2C8 and GAPDH (17). The selected TAA were also found to be expressed in the tumor tissue of a large cohort of PDA patients from which the cohort employed in this study was derived (22; **Supplementary Figure 2**). In this article, we have characterized the profiling of T lymphocyte responses to the same TAA panel in a different cohort of PDA patients treated primarily with GEM-based CT. We observed that CT shapes the cellular response and clonal expansion of T lymphocytes against TAA and influences the gene expression involved in metabolic pathways in about half of the patients, showing an *in vitro* T lymphocyte effector response after CT. These findings support the fact that combining TAA stimulation with CT could be an effective immunotherapeutic strategy for PDA (this study and (17)). This evidence is suggested by the observation that, in this patient cohort, the *in vitro* stimulation of T lymphocytes with the same TAA in the presence of immune checkpoint inhibitors did not induce a shift toward an effector response either before or after CT (data not shown). This finding is consistent with data from clinical trials in which checkpoint inhibitors did not increase survival in PDA patients (23–25).

Analyzing the proliferative response of TAA-stimulated T lymphocytes demonstrated that CT did not induce significant alterations in the intensity or in the number of proliferative responses. Conversely, analyzing immunological tone revealed that this was shifted from regulatory to effector response in around 50% of the patients after CT.

Interestingly, T lymphocyte proliferation in response to at least one TAA, either before or after CT, was observed in almost all patients, and the number of responsive TAA correlated with higher overall survival. The analyzed TAA, which are typically overexpressed in PDA (17, 26–30), are suggested to be potential immunotherapeutic targets even in advanced PDA patients. In particular, two of these TAA that are involved in the glycolytic pathway (ENO1 and GAPDH) are able to elicit an antitumor effector response (26–28, 30).

We observed that the increase in the number of TAA recognized by PDA patients after CT could be due to an antigen spreading effect, as in patient sera the presence of circulating antibodies against a number of antigens, including those employed in this study, was increased by CT treatment (17).

The shift of quality of the anti-tumor response toward an effector immunological tone, could be due to the induction of immunogenic cell death (ICD) upon CT treatment (31, 32). In fact, ICD alters TAA expression from dying cancer cells, alerting the immune system, and this process activates T lymphocytes, particularly CD8+ cytotoxic T cells, which play a key role in attacking cancer cells through cytokine production such as IFN-γ (33, 34). The impact of CT in shifting the T lymphocyte response toward an effector phenotype was also confirmed by the decrease - after CT - in IL-10 production from TAA-stimulated T lymphocytes, which allowed the attenuation of the regulatory phenotype. It is well established that certain cytokines can be produced by antigen-presenting cells, including IL-10, IL-12 and TGFβ. Consequently, it is not possible to distinguish the contribution of each subset of the peripheral blood cells to cytokine production. In contrast, other cytokines, such as IFN-γ and IL-17, are predominantly secreted by T lymphocytes. Our data also show that after CT a fraction of TAA-induced responses are deflected to Th17-like effector response, whereas a limited number of TAA-induced responses are characterized by IL-12 increase and consequently contribute to the high IFN-γ production.

The CT-induced antitumor effector response was seen in about 50% of PDA patients, with the patients remaining resistant to CT. In some patients the T lymphocyte effector response to TAA was displayed only before CT, shifting to the regulatory one after CT and indicating their exhausted phenotype (35).

As a model to study the influence of CT on the TCRB repertoire, we evaluated the effect of stimulation with ENO1 on T lymphocytes from PDA patients. We demonstrated that CT modifies the TCRB repertoire of ENO1 specific T cell clones in almost all patients analyzed. In some patients CT induced ex novo or enhanced ENO1-specific T cell clones. These data strongly suggest that CT reshapes the TAA-specific TCR repertoire by decreasing or eliminating clonotypes that were present before CT and inducing *de novo* or inducing TAA-specific clonotypes. Nevertheless, as after CT sequencing data revealed no significative differences in the immune cell subset distribution, it cannot be excluded that CT facilitates the elimination of some TCR clonotypes, independently of TAA-stimulation.

Since the analysis of TCRB was performed on peripheral T lymphocytes, the impact of CT on the TCRB repertoire of infiltrating T lymphocytes that infiltrate PDA was not evaluated. However, previous studies on T cell clones from PDA patients, showed that ENO1-specific T cell clones generated from peripheral blood or tumor biopsies displayed the same TCRB repertoire, suggesting that they can recirculate from the tumor and periphery (36).

The stratification of PDA patients based on both the proliferative response and immunological tone revealed a better survival rate in the groups in which after CT there was an increase in TAA-specific proliferation (i.e., “CT responder”) and an increase in TAA-induced effector phenotype (i.e., “CT effector”), supporting the hypothesis that the anti-tumor immune response to these TAA is associated with a better outcome. In addition, a multi-variate analysis considering age, metastasis and surgery did not show any statistically significant association with the TAA response (data not shown).

The analysis of transcriptomics profiles of PBMC from PDA patients after CT showed that “CT exhausted/regulatory” patients display a higher expression of IL1R antagonist related genes (IL1R2, IL1RN). This observation is consistent with the expression levels of IL1A, IL1RN, IL1R2, and IL1RAP, which were increased in PDA tumor tissue and decreased in PDA patients with a better survival (37). In addition, in PDA orthotopically injected mice, IL-1R antagonist inhibited the secretion of thymic stromal lymphopoietin (TLP) by cancer-associated fibroblasts (38). TSLP favored Th2 cell polarization, which in PDA patients is associated with reduced survival. By contrast, in immunodeficient tumor-bearing mice, the administration of polarized Th2 cells reduced gastrointestinal tumor growth, including PDA (39); since cytokines and immune cells could exhibit dual functions in cancer, Th2 immunity deserves further investigation.

We showed that the expression of LDAH, a serine hydrolase activity protein involved in lipid metabolism and nutrient availability (40, 41) was higher in “CT effector” patients after CT. The expression of LDAH was prevalently associated with the presence of CD4+ T lymphocytes, suggesting that lipid metabolism in this cell subset can influence their antitumor functional activity. Notably, LDAH was found upregulated in tumor tissue of PDA patients (37, 42), leading to a connection between our observation in peripheral blood and the tumor.

Overall, the gene expression of IL1R related genes and LDAH could be useful markers to predict the outcome of PDA patients to CT in combination with TAA vaccination.

## Data availability statement

The original contributions presented in the study are publicly available. This data can be found here: BioProject, accession number PRJNA1144662.

## Ethics statement

The studies involving humans were approved by A.O.U. Città della Salute e della Scienza di Torino, Torino, Italy. The studies were conducted in accordance with the local legislation and institutional requirements. The participants provided their written informed consent to participate in this study.

## Author contributions

SiB: Data curation, Formal analysis, Writing – original draft, Writing – review & editing. SaB: Data curation, Formal analysis, Writing – original draft, Writing – review & editing. CC: Formal analysis, Funding acquisition, Writing – review & editing. MA: Formal analysis, Writing – review & editing. RC: Formal analysis, Writing – review & editing. LB: Methodology, Writing – review & editing. EG: Methodology, Writing – review & editing. RS: Investigation, Resources, Writing – review & editing. MS: Investigation, Resources, Writing – review & editing. DC: Resources, Writing – review & editing. DG: Data curation, Resources, Writing – review & editing. PC: Conceptualization, Funding acquisition, Writing – review & editing. FC: Conceptualization, Formal analysis, Resources, Writing – review & editing. FN: Conceptualization, Data curation, Funding acquisition, Investigation, Project administration, Supervision, Writing – original draft, Writing – review & editing.
