# Supplementary figures and images for: In pancreatic cancer patients, chemotherapy reshapes the gene expression profile and antigen receptor repertoire of T lymphocytes and enhances their effector response to tumor-associated antigens
Source: Front Immunol. 2024 Aug 8;15:1427424. doi: 10.3389/fimmu.2024.1427424 (PMC11339620; doi:10.3389/fimmu.2024.1427424)

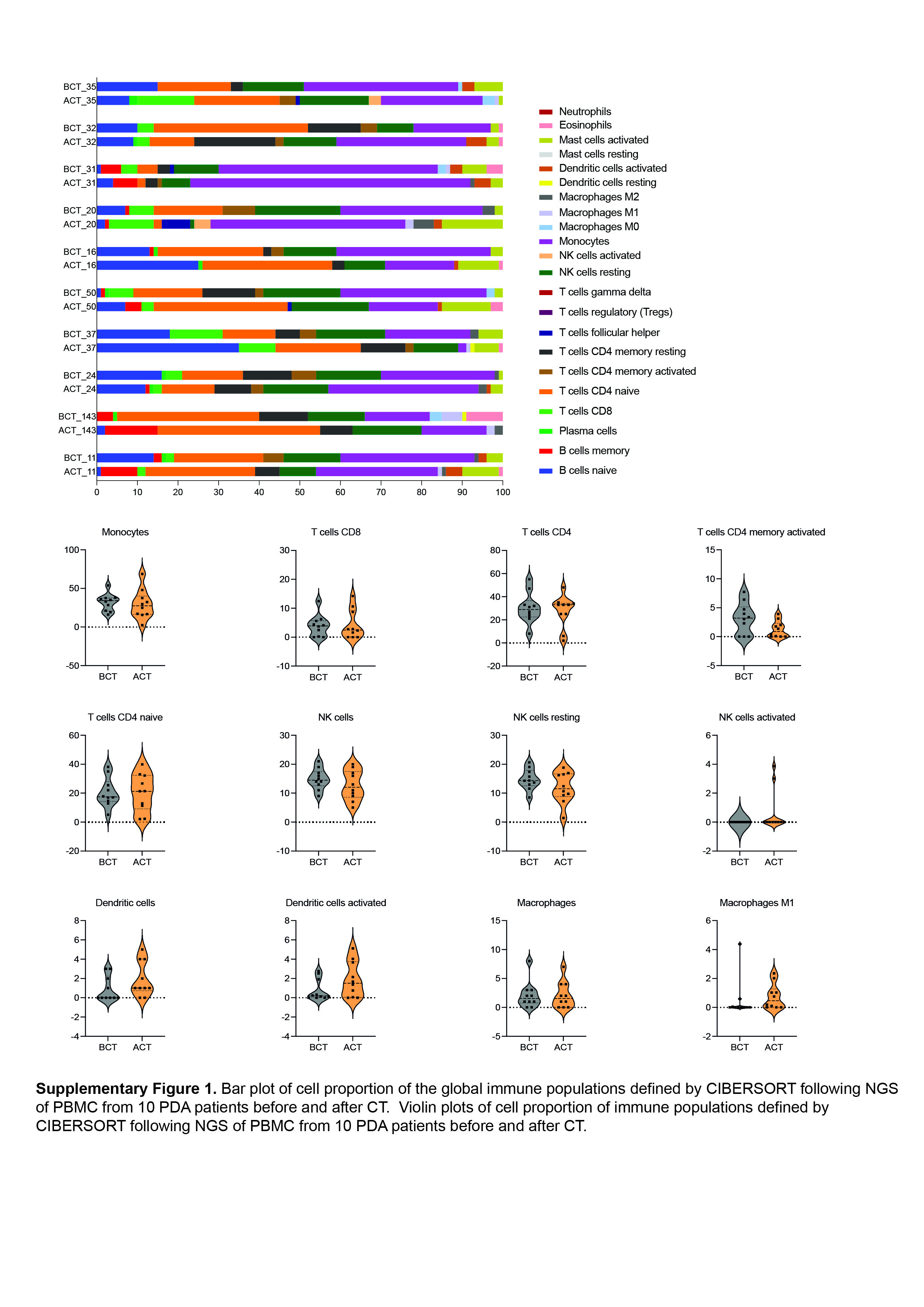

Supplement: Supplementary file 1 [file Image_1.jpg]

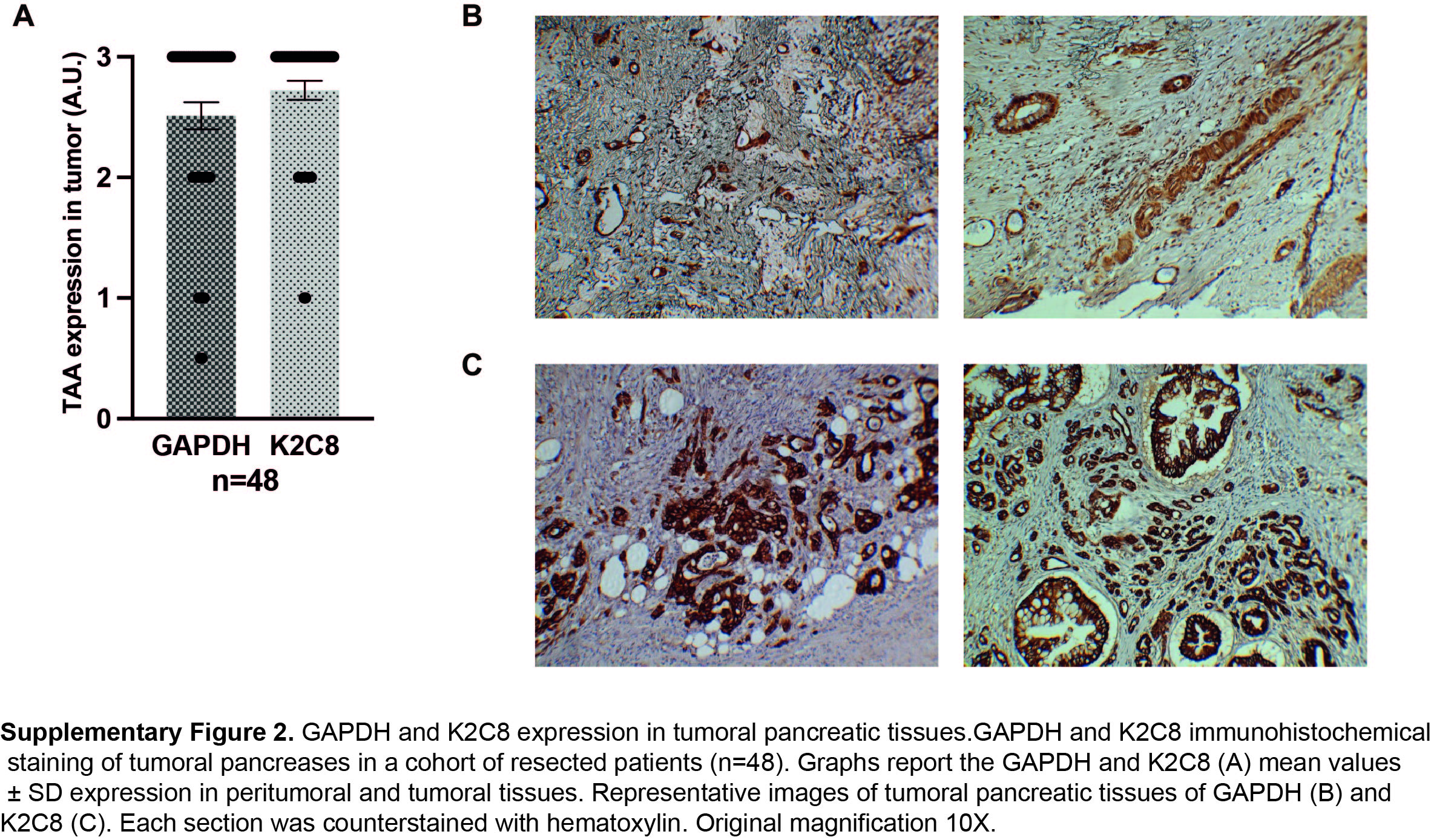

Supplement: Supplementary file 2 [file Image_2.jpeg]
